# Organic and inorganic fertilizers modulate the response of the soil microbiome to salinity stress

**DOI:** 10.3389/fmicb.2025.1551586

**Published:** 2025-06-19

**Authors:** Halima Malal, Joshua A. Garcia, Anna Marrs, Mohamed Ait Hamza, Courtney Emerson, Mallika Nocco, Hicham Lakhtar, Cristina Lazcano

**Affiliations:** ^1^Microbial Biotechnology and Plant Protection Laboratory, Faculty of Science, Ibn Zohr University, Agadir, Morocco; ^2^Department of Land, Air and Water Resources, University of California, Davis, Davis, CA, Unites States; ^3^Laboratory of Biotechnology and Valorization of Natural Resources, Faculty of Sciences, University of Ibn Zohr, Agadir, Morocco; ^4^Department of Biological Systems Engineering, University of Wisconsin-Madison, Madison, WI, United States

**Keywords:** soil salinization, vermicompost, NPK fertilizer, soil multifunctionality, bacterial network

## Abstract

Salinity stress threatens soil microbiomes, a key driver of soil multifunctionality and health. This study employed high-throughput sequencing of 16S rRNA, PLFAs, multifunctionality index, and co-occurrence networks to gain a comprehensive understanding of the dynamic responses of soil microbiomes to salinity stress gradient (0, 0.4 and 1 mol NaCl). Additionally, we investigated how these responses are shaped by the addition of vermicompost and NPK fertilizer during short-term (2-h) and long-term (70-day) incubation periods. Salinity stress reduced bacterial and fungal phospholipid fatty acids (PLFA) concentrations in the short-term. Over the long-term, the microbial community evolved into a new pattern under salt stress, favoring the presence of *Bacteriodota*, a salt-tolerant phylum, while decreasing the relative abundance of *Acidobacteriota* and *Planctomycetota*, which are more salt-sensitive. Furthermore, salinity decreased species richness by 11.33% and soil multifunctionality by 21.48% but increased microbial network complexity while decreasing its stability. Incorporating vermicompost increased bacterial and fungal PLFAs, enhanced bacterial diversity by 2.33%, promoted salt-tolerant bacteria, and increased the complexity and stability of the bacterial network. Conversely, the application of NPK fertilizer reduced bacterial richness, alpha diversity and soil multifunctionality by 14.52, 5.83, and 12.34%, respectively, further disrupting the microbial community and making resilience to salinity stress more challenging. Furthermore, NPK fertilization increased bacterial network complexity but decreased its stability. This study underscores the significance of employing vermicompost to improve the health of saline soils. Furthermore, it emphasizes the negative impacts of NPK fertilizer on soil microbial structure and function and hinder its recovery from salinity’s impacts.

## 1 Introduction

Soil salinization is a global challenge stemming from natural and human-induced factors, particularly affecting arid and semi-arid regions due to high temperatures, limited rainfall, and intensive agriculture practices (Ait-El-Mokhtar et al., 2020; Benazzouk et al., 2020). This phenomenon threatens over 11% of irrigated soils in such areas, with projections of a 40% increase due to climate change and intensified agriculture (Garcia-Franco et al., 2021). Given that nearly 40% of global food production occurs in these regions, soil salinization poses a significant threat to global food security, causing an estimated annual loss of 124 trillion kilocalories in agricultural productivity (Garcia-Franco et al., 2021; Prasertsuk and Wijitkosum, 2021).

The soil microbiome is pivotal in preserving soil health and supporting crop productivity by facilitating essential soil functions including organic matter decomposition, nutrient cycling, and carbon sequestration (Jiang et al., 2019). Moreover, rich and diverse microbial networks enhance soil multifunctionality (Wagg et al., 2019), defined as the ability of the soil to perform multiple functions simultaneously (Delgado-Baquerizo et al., 2016). Additionally, healthy soils are characterized by higher soil multifunctionality and are more resilient to environmental stressors (Luo et al., 2023).

Salinity stress can profoundly affect the soil microbiome (Fierer et al., 2021). Increased osmotic pressure and ion toxicity reduce microbial biomass, activity, and diversity, exerting strong selective pressure on the microbial community (Haj-Amor et al., 2022). These alterations can disrupt nutrient cycling, impair soil metabolic activity, and decrease soil multifunctionality (Cheng and Wan, 2023; Jia et al., 2023), ultimately jeopardizing soil health and crop production (Abdul Rahman et al., 2021; Yan et al., 2015).

The soil microbiome can undergo interactive responses to salinity stress when subjected to agricultural practices. Conventional use of synthetic fertilizers reduces microbial richness, diversity, and soil multifunctionality, negatively impacting soil health (Castellano-Hinojosa et al., 2021). Conversely, organic fertilizers increase bacterial diversity and richness, promoting functional taxa involved in heterotrophic metabolism and nitrogen fixation, thereby enhancing soil multifunctionality and microbial network complexity (Maji et al., 2017; Gu et al., 2019; Hubanks et al., 2018). Previous studies have indicated that applying an organic fertilizer can alleviate the impacts of salinity on the soil microbiome by enhancing microbial richness and providing essential nutrients and organic matter (Mao et al., 2022; Wichern et al., 2020). However, our understanding of the impact of organic and inorganic fertilizers on the resilience of the soil microbiome to salinity stress over time remains limited. Furthermore, it remains unclear how the interactions between salinity and fertilizer type impact soil microbiome structure, diversity, network complexity, and soil multifunctionality.

Therefore, this study aimed to investigate these dynamics by combining the PLFA and 16S rRNA sequencing analysis. While PLFA analysis offers insights into the microbial community structure and biomass by identifying the lipid profiles of various microbial groups (Buyer et al., 2019), the 16S rRNA sequencing provides detailed taxonomic information at a finer resolution, identifying specific bacterial taxa and their relative abundances (Orwin et al., 2018). Combining these methods will allows us to capture both broad and specific changes in microbial communities.

In this study we aimed to (1) examine the immediate response of the soil microbiome to salinity stress through a short-term incubation study and its recovery from the induced stress after 70 days of its application, (2) identify the impact of salinity on microbial structure and diversity, microbial network, and soil multifunctionality, and (3) to identify the impact of vermicompost and NPK fertilizer on the response of soil microbiome to salinity stress. We hypothesized that salinity stress would initially reduce microbial abundance, diversity, and functions, but recover in time dependent manner. Additionally, we hypothesized that the application of vermicompost would enhance microbial structure and function, mitigating the negative effects of salinity stress. This enhancement is expected to occur through increased nutrient availability and the enrichment of specific microbial taxa present in vermicompost. In contrast, we hypothesized that chemical fertilizers would reduce microbial functions and diversity, exacerbating the impact of salinity stress on the soil microbiome. This detrimental effect may result from nutrient imbalances and reduced organic matter inputs, which negatively affect microbial community composition and lower microbial biomass and diversity.

## 2 Materials and methods

### 2.1 Fertilizers and soil

The organic fertilizer used in this study was vermicompost collected from a vermifilter system situated at a commercial dairy in Hilmar, California (United States) and operated by BioFiltro Inc. The vermifilter functions as a wastewater treatment system for dairy wastewater, primarily composed of liquid manure. The system employs a 0.5 m layer of woodchips as a bedding material, inoculated with *Eisenia fetida* earthworms, typically inhabiting the top 30 cm of the system. Analysis of the vermicompost revealed key physicochemical characteristics, including a total C content of 371 g kg^–1^ and a total N content of 29 g kg^–1^. The pH of the vermicompost was 7.4, and its electrical conductivity (EC) was 3.17 ds m^–1^.

The soil used in this study was collected from the top 0–15 cm of an agricultural field under commercial production in Five Points, California, United States in 2022. The soil is a Posochanet clay loam, saline-sodic, wet, 0–1% slopes (University of California Davis, n.d.). After collection, the soil was sieved to an 8 mm particle size, homogenized, stored moist in covered bins at 4°C, and subsequently air dried for a week prior to use in the incubation experiments. The soil had a total C content of 9.17 g kg^–1^ and a total N content of 1.29 g kg^–1^. The soil’s pH was 7.16 and electrical conductivity was 2.18 ds m^–1^.

### 2.2 Experimental design

We conducted two soil incubation experiments (short- and long-term), using a complete randomized design with two factors: fertilizer type and salinity level. Each factor had four replicates. The fertilizer types used were (1) vermicompost, (2) an equivalent amount of N, P, and K provided as inorganic fertilizer, and (3) a control group with no fertilizer added. We applied these fertilizer treatments to 120 mL specimen cups, each containing 80 g of air-dried soil. The vermicompost treatment received 1.92 g of moist vermicompost, which equated to 371 g C Kg^–1^ of soil, 29 g N Kg^–1^ of soil, 0.69 g PO_4_^2–^ Kg^–1^ of soil, and 1.15 g K Kg^–1^ of soil. This application rate was chosen based on recommended rates for California croplands and the vermicompost’s C:N ratio (Gravuer and Guanasekara, 2016). We used a blend of urea, calcium dihydrogen phosphate, and potassium sulfate for the inorganic fertilizer treatment to provide the same amount of total N, P, and K as the vermicompost treatment. All treatments, including the control, were thoroughly mixed, and we adjusted the water content to reach 60% of the soil’s water holding capacity determined by the capillarity method. To calculate water holding capacity, the initial weight of the soil sample was measured, and the sample was saturated with water. After excess water was drained off, the sample was reweighed to determine the amount of water retained (Haney and Haney, 2010).

In the short-term incubation experiment, we investigated the soil microbiome’s response to salinity stress when different fertilizers were present. This was done by assessing the impact of the treatments on the diversity and function of the soil microbiome after 2 h of salinity application. This incubation time was chosen based on previous evidence of the rapid response of the soil microbiome to salinity, which can be detected within a few hours of its application, as demonstrated by Rath et al. (2016) and Bursy et al. (2008).

Each specimen cup containing 80 g of soil and a specific fertilizer treatment was placed in a 0.5 L mason jar. Five mL of deionized (DI) water was added to the jar to maintain moisture of the air inside the jar. The mason jars were closed with lids featuring a small hole at the top, covered with a piece of cotton for gas exchange. Prior to salinity treatment, the soils underwent a 10-day preincubation period at 25°C in the dark to equilibrate the soil microbial community in the presence of the different fertilizers and minimize any potential pulse effect.

Following this preincubation period, we introduced salinity stress using NaCl solution at varying concentrations, resulting in three distinct salinity levels: no salinity (0 mol NaCl), medium salinity (0.4 mol NaCl), and high salinity (1 mol NaCl). These salinity levels were selected based on the work of Szoboszlay et al. (2019) and to reflect the salinity challenges in agricultural fields in the western San Joaquin Valley. We adjusted the volume to reach 60% of the water holding capacity during incubation. The combination of salinity stress and the different fertilizer treatments resulted in nine distinct experimental conditions (Table 1).

**TABLE 1:** Salinity and fertilizer treatments applied to the soil in the short- (2 h) and long-term (70 days) incubation experiments.

Treatment	Salinity	Vermicompost	NPK
1	Control	– (No fertilizer)	– (No fertilizer)
2	Control	+	–
3	Control	–	+
4	Medium	– (No fertilizer)	– (No fertilizer)
5	Medium	+	–
6	Medium	–	+
7	High	– (No fertilizer)	– (No fertilizer)
8	High	+	–
9	High	–	+

The mason jars containing the soil samples were then placed in a dark environment at a constant temperature of 25°C for a period of 2 h, following the methodology established by Rath et al. (2016). After the 2-h incubation period, soil samples were extracted from the specimen cups. A subsample was promptly collected and stored at –80°C for subsequent analysis of 16S rRNA, phospholipid fatty acid (PLFA), and enzyme activities. The remaining portion of the soil was placed in plastic bags and stored at 4°C for further biochemical analyses.

For our long-term incubation experiment, soil samples were incubated in 0.5 L Mason jars under controlled aerobic conditions for 70 days. To enable continuous monitoring of microbial respiration during this extended incubation, we sealed the Mason jars airtight and incorporated two septa in the lids. We maintained the soil’s moisture content at 60% of its water holding capacity throughout the incubation period by periodically adding sterile deionized water as needed. Upon the conclusion of the 70-day incubation, we destructively sampled the soils adhering to the same procedure outlined previously for the short-term incubation.

### 2.3 Measurement of soil multifunctionality

For the multifunctionality assessment, we quantified 13 soil functions related to the carbon, nitrogen, and phosphorus cycles. These functions included microbial biomass carbon (MBC), mineralizable carbon, total carbon, cellulase enzyme activity (CB), α-glucosidase enzyme activity (AG), β-glucosidase enzyme activity (BG), and xylosidase enzyme activity (XYL) for the carbon cycle; nitrate (NO_3_^–^-N), ammonium (NH_4_^+^-N), total nitrogen, leucine aminopeptidase enzyme activity (LAP), and N-acetyl-glucosaminidase enzyme activity (NAG) for the nitrogen cycle; and phosphatase enzyme activity (PHOS) for the phosphorus cycle. The multifunctionality index was calculated using the averaging approach after *Z*-score transformation, calculated using the multifunc package in R software (Byrnes et al., 2014).

Nitrate (NO_3_^–^-N) and ammonium (NH_4_^+^-N) concentration were determined colorimetrically in soil samples. We prepared a soil extract with 8 g of fresh soil using 0.5 M K_2_SO_4_, following the methods of Doane and Horwath (2003) and Miranda et al. (2001). To determine the concentration of MBC, we subjected a subsample of 6 g to chloroform fumigation for 24 h, followed by an extraction with 30 mL of 0.5 K_2_SO_4_. Another 6 g subsample was used to prepare a non-fumigated extract. The concentrations of dissolved organic carbon were determined by UV-persulfate oxidation (Teledyne-Tekmar Fusion), with the MBC defined as the difference between the fumigated and non-fumigated samples (Horwath and Paul, 1994). Enzyme activity analysis was conducted via a high-throughput microplate with fluorescence detection, following the protocol established by Bell et al. (2013). Soil respiration was estimated over a 70-day incubation period by measuring CO_2_ evolution from samples using LICOR 850 IRGA CO_2_/H_2_O analyzer (LI-COR Environmental, Lincoln Nebraska, United States). The jars were opened for 5 min after each measurement to equilibrate CO_2_ and H_2_O gasses. Measurements were taken every 2 days, and cumulative respiration was calculated. Total soil C (%) and N (%) were determined by dry combustion in an elemental analyzer (Costech analytical technologies Inc. model ECS 4010).

### 2.4 Analysis of the soil microbial community structure

We evaluated the effects of salinity and fertilizer treatments on the soil microbial community structure using two analytical approaches: phospholipid fatty acid (PLFAs) profiling and high-throughput sequencing of prokaryotic markers (16S rRNA).

For the PLFA analysis, soil samples were shipped to Regen Ag Lab (Pleasanton, NE, United States). The samples underwent extraction, fractionation, and transesterification before analysis using a 7890-gas chromatograph (GC) equipped with a 7693 autosampler, split-spitless inlet, and flame ionization detector (FID) from Agilent, Wilmington, DE, United States. The GC-FID system was controlled using Agilent ChemStation and MIDI’s Sherlock software. PLFAs were assigned to various microbial groups, including total living microbial biomass (TLMB), total bacteria (TB), total fungi (TF), Gram-positive bacteria (GP), Gram-negative bacteria (GN), Actinomycetes, arbuscular mycorrhizal fungi (AMF), and saprophytic fungi, and the results were reported in nanograms per gram of dry soil. The biomarkers i15:0, a15:0, i16:0, 16:1ω7c, i17:0, a17:0, cy17:0, 18:1ω7c, cy19:0 were used for total bacteria. The biomarkers a15:0, i15:0, i16:0, a17:0, i17:0 were used for GP, and cy17:0, 18:1ω7c, cy19:0, 16:1ω7c for GN. The biomarker 10Me18:0 was used for actinomycetes. 18:2ω6,9 was used for saprophytic fungi, and 16:1ω5 was used for AMF, while 18:2ω6c was used for total fungi (Frostegård and Bååth, 1996; Ngosong et al., 2010; Zelles, 1999).

High throughput sequencing of prokaryotic markers (16s rRNA) was conducted at the Genome Center DNA Technologies Core of the University of California, Davis. Initially, DNA was extracted from 1 g of frozen soil (-80°C) using the Qiagen PowerSoil Pro Kit (Catalog No. 47016, Qiagen, Germany). The V4 domain of the 16S rRNA was amplified using primers 515F and 806R in a two-step PCR procedure. In the first step, PCR conditions involved an initial incubation at 95°C for 3 min, followed by 30 cycles of 95°C for 45 s, 50°C for 30 s, 72°C for 30 s, and a final extension at 72°C for 3 min. In the second step, PCR conditions included an initial incubation at 95°C for 3 min, followed by 9 cycles of 95°C for 30 s, 58°C for 30 s, 72°C for 30 s, and a final extension at 72°C for 3 min. The resulting product was quantified using the Qubit High Sensitivity dsDNA kit (Invitrogen), and individual amplicons were combined in equal concentrations. The pooled library was purified using Ampure XP beads (Beckman Coulter) and was assessed for quality and proper amplicon size on an Agilent 2100 Bioanalyzer (Agilent Technologies). The library’s concentration was determined via qPCR followed by 300-bp paired-end sequencing on an Illumina MiSeq instrument (Illumina).

Subsequently, the raw sequencing data were processed using QIIME2-2022.11 to cluster OTUs and generate an OTU table and a taxonomy table (Bolyen et al., 2019). The analysis began with importing the paired end demultiplexed FASTQ files to QIIME using a manifest file. A denoising step was performed on the sequence data using a DADA2 to remove lower quality portions of the read segments. OTUs were then clustered using *de novo* OTU-clustering at 97% similarity threshold, with chimera filtering carried out using Uchime. Taxonomy assignment for each OTU was accomplished using the Naïve Bayes Classifier, which was trained on the weighted Greengenes 13_8 99% database for sequence from the 515F/806R region (Kaehler et al., 2019). The resulting sequences were grouped into 37944 OTUs for the short-term incubation and 31414 OTUs for the long-term incubation, spanning 38 phyla, 114 classes, 163 orders, 227 families, 233 groups, and 81 species. To assess diversity, Shannon diversity indices was calculated for each sample using the diversity function. The ACE species richness index was calculated using the estimate function, while evenness was determined using both diversity and specnumber functions.

### 2.5 Statistical analysis

We used R software (version 4.2.2) for statistical analysis (R Core Team, 2022), conducting a two-way ANOVA to assess salinity and fertilizer effects on PLFAs concentrations measured in the soil. Normality and homogeneity of data were checked using the Shapiro-Wilk test for normality and Levene’s test for homogeneity of variances, both implemented through the check_model function from the “car” package in R software. The box-cox transformations were applied for variables that did not meet the assumptions. Tukey tests were used for mean comparisons.

For graphical representation of statistical results, we used a dual-letter notation system to differentiate between main effects. Uppercase letters were used to compare fertilizer treatments within each salinity level, while lowercase letters were used to compare salinity treatments within each fertilizer type. For variables where a significant interaction between salinity and fertilizer was detected lowercase letters were used to compare all treatment combinations.

Microbiome analysis included Bray-Curtis distance calculation, PCoA visualization, and PERMANOVA. Linear models, ANOVA, and Tukey tests examined diversity responses to salinity and fertilizer. We used phyloseq to aggregate data, identify abundant phyla, and conducted ANOVA to compare their relative abundance. To identify enriched families (Segata et al., 2011), we employed Microbiome Analyst’s LEfSe method (Dhariwal et al., 2017). We used random matrix theory (RMT) and Spearman correlation to study microbial co-occurrence networks (Feng et al., 2022). The networks were visualized in Gephi, and the network topologies were analyzed using methods outlined in Ji et al. (2021).

## 3 Results

### 3.1 Impact of salinity and fertilizers application on microbial community structure

#### 3.1.1 Short term incubation experiment

**FIGURE 1:**
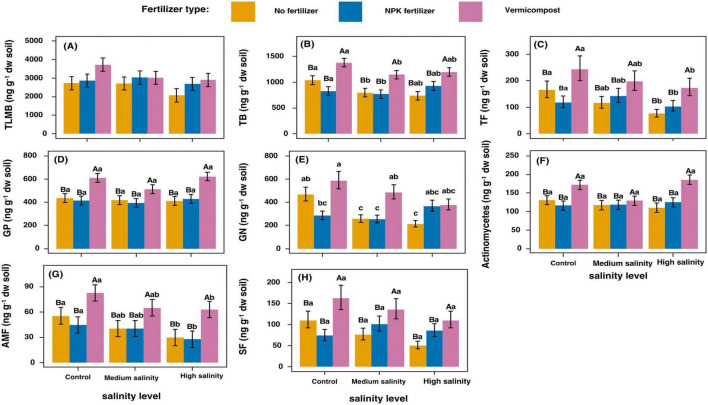
Changes in phospholipid fatty acid (PLFA) concentrations in soil samples 2 h after treatments application. Values represent means of 4 replicates ± SD. **(A)**: No significant effect was detected. **(B–D,F–H)**: For each fertilizer treatment (across salinity levels): means with the same uppercase letters are not significantly different (*p* < 0.05). For each salinity treatment (across fertilizer types): means with the same lowercase letters are not significantly different (*p* < 0.05). **(E)** For each combination of fertilizer type and salinity level, means followed by the same lowercase letters are not significantly different at *p* < 0.05. TLMB, total living microbial biomass; TB, total bacteria; TF, total fungi; GP, Gram-positive bacteria; GN, Gram-negative bacteria; AMF, arbuscular mycorrhizal fungi; SF, saprophytic fungi.

Salinity significantly decreased total bacteria (*P* < 0.05) between medium and control treatments (Figure 1B). Higher salinity levels reduced the concentration of GN, TF, and AMF compared to control conditions (Figures 1C,E,G). The addition of vermicompost significantly increased the abundance of all measured microbial groups compared to the no fertilizer treatment (Figures 1B–H). The most substantial increases were observed in SF and AMF, while more moderate increases occurred in GP and actinomycetes (Figures 1A–H).

Increasing salinity increased the ratio of GP/GN bacteria from 1.19 ± 0.01 in the no fertilizer treatment to 2.43 ± 0.30 in the high salinity treatment (Supplementary Table S1). Vermicompost application decreased the ratio saturated to unsaturated PLFAs from 4.22 ± 0.81 in the no fertilizer treatment to 3.00 ± 0.84 in the vermicompost treatments.

Predominant bacterial phyla (relative abundance > 3%) included *Proteobacteroidota* (30%), *Planctomycetota* (11%), *Bacteroidota* (9%), *Actinomycetota* (9%), *Chloroflexota* (8%), *Bacillota* (5%), *Gemmatimonadota* (5%), *Acidobacteriota* (3%), collectively accounting for 81% of the bacterial sequences (Supplementary Figures S1A,B).

Shannon alpha diversity and ACE index remained stable across salinity and fertilizer treatments after 2 h of incubation (Figures 3a,b). However, bacterial community composition shifted, as depicted in the PCoA graph (Figure 4A). Vermicompost had a distinct bacterial community and formed a separate cluster from inorganic fertilizer and no fertilizer treatments. PERMANOVA analysis identified fertilizer type as the only significant factor shaping the bacterial community (*R*^2^ = 0.20, *p* < 0.001).

ANOVA results for the top eight most abundant bacterial phyla (Supplementary Table S2) indicated that the relative abundances of *Pseudomonadota, Planctomycetota, Actinomycetota, Gemmatimonadota, Bacillota*, and *Acidobacteriota* were not significantly affected by either salinity stress or fertilizer application (*p* > 0.05). However, the application of vermicompost increased the relative abundance of *Bacteriodota* from 6.29 ± 0.79 in the no fertilizer treatment to 7.75 ± 1.44. Additionally, *Chloroflexota* abundance decreased slightly with vermicompost application compared to the no fertilizer treatment.

Furthermore, LEfSe analysis revealed that the prokaryotic families *Cryomorphaceae, Nakamurellaceae, Hyphomicrobiaceae, Xanthomonadaceae, Rhodobacteraceae, Gordoniaceae, Mycobacteriaceae, Chitinophagaceae* were enriched in vermicompost-treated soils (Supplementary Figure S2).

#### 3.1.2 Long-term incubation experiment

**FIGURE 2:**
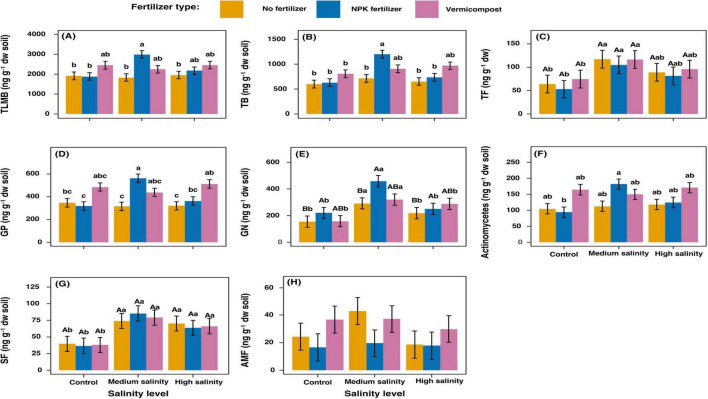
Changes in phospholipid fatty acid (PLFA) concentrations 70 days after treatments application. Values represent means of 4 replicates ± sd. **(E,G)** For each fertilizer treatment (across salinity levels): means with the same uppercase letters are not significantly different (*p* < 0.05). For each salinity treatment (across fertilizer types): means with the same lowercase letters are not significantly different (*p* < 0.05). **(A–D,F)** For each combination of fertilizer type and salinity level, means followed by the same lowercase letters are not significantly different at *p* < 0.05. **(H)**: No significant effect was detected. TLMB, total living microbial biomass; TB, total bacteria; TF, total fungi; GP, Gram positive bacteria; GN, Gram negative bacteria; AMF, Arbuscular Mycorrhizal Fungi; SF, Saprophytic Fungi.

Medium salinity stress substantially increased TB, GN, TF, and SF relative to the control (Figures 2B,C,E,G). However, this effect was reversed under high salinity conditions, resulting in PLFAs concentrations similar to those under control salinity levels (Figures 2B,C,E,G).

**FIGURE 3:**
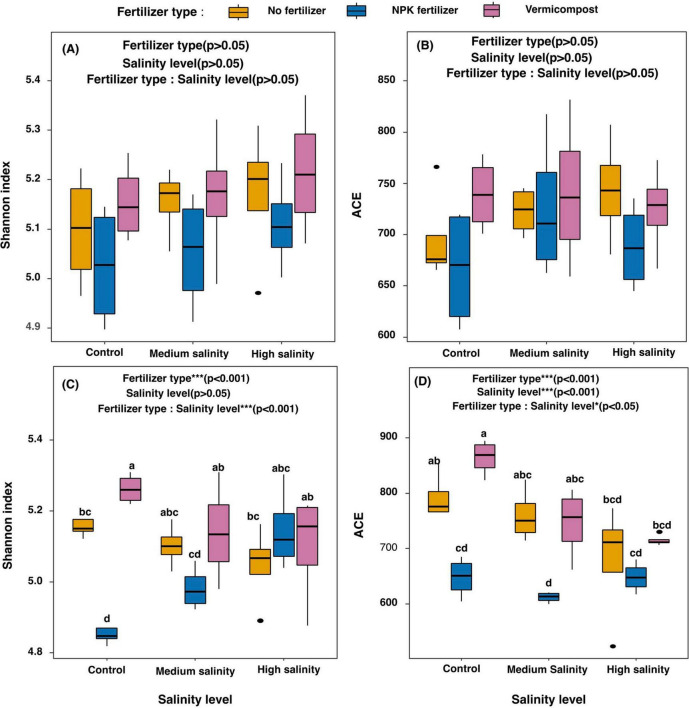
Changes in Shannon, ACE indexes of soil samples along the salinity gradient and in different fertilizer types, 2 h **(A,B)** and 70 days **(C,D)** after the start of the incubation. For each combination of fertilizer type and salinity level, means followed by the same lowercase letters are not significantly different at *p* < 0.05 according to Tukey’s test results. The results of the two-way ANOVA are presented at the top of each graph.

**FIGURE 4:**
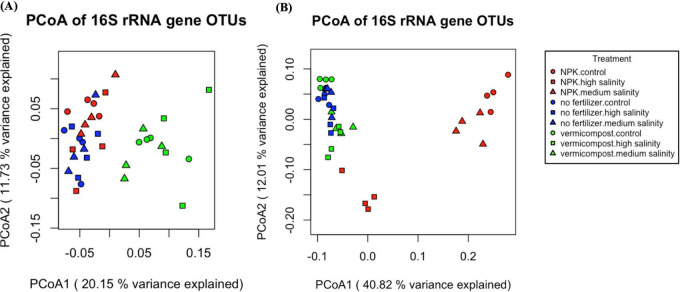
Principal coordinates analysis (PCoA) of soil microbial community based on 16 S rRNA gene sequencing after a 2 h incubation **(A)** and a 70-day incubation **(B)**. Fertilizer type* salinity level treatment groups had at least 4 replicates, in total sequences from 36 samples were displayed in this analysis.

The use of vermicompost enhanced several microbial PLFAs compared to the no fertilizer treatment, with moderate increases in TLMB and TB, and slightly larger increases in GP and actinomycetes (Figures 2A,B,D,F).

Inorganic fertilizer had a strong positive effect under medium salinity, significantly increasing most bacterial markers compared to control conditions. This effect was particularly pronounced for GN bacteria and actinomycetes. However, this effect was reversed in high salinity, bringing PLFAs to the same level as the control salinity (Figures 2A,B,D–F).

The salinity level significantly influenced the GP/GN ratio (*p* < 0.001), leading to a decrease from 3.31 ± 1.08 in the control to 1.69 ± 0.49 in medium salinity and 2.2 ± 0.53 in high salinity. Salinity stress affected significantly the Saturated/Unstaturated ratio (*p* < 0.001). The ratio decreased from 7.51 ± 3.09 in the control to 3.46 ± 1.00 in medium salinity and 4.73 ± 1.27 in high salinity (Supplementary Table S1).

Under control salinity conditions, the application of vermicompost increased slightly the Shannon diversity index, while the use of NPK fertilizer decreased it compared to the no-fertilizer treatment. In contrast, under high salinity, the application of NPK fertilizer increased the Shannon index compared to the control salinity (Figure 3C) while no effects of vermicompost were observed at high salinity.

The ACE index dropped from 864 under control salinity to 715 under high salinity (*p* < 0.001). Furthermore, inorganic fertilizer application reduced bacterial richness by 18 and 19% under control and medium salinity conditions, respectively, compared to the no-fertilizer treatment (*p* < 0.05) (Figure 3D).

The PERMANOVA analysis showed significant effects of salinity level (*R*^2^ = 0.14, *p* < 0.001), fertilizer type (*R*^2^ = 0.37, *p* < 0.001), and their interaction (*R*^2^ = 0.18, *p* < 0.001) on bacterial community composition. The addition of inorganic fertilizer disrupted the bacterial community the most, especially in the control and medium salinity, with points corresponding to those treatments clustering together, separated from high salinity treatments. Vermicompost and no fertilizer treatments tended to cluster together, with vermicompost treatments categorized by salinity level (Figure 4B).

Changes in the relative abundance of the predominant bacterial phyla under salinity and fertilizer treatments were observed (Supplementary Table S3). Salinity stress significantly decreased the relative abundance of *Planctomycetota* under both medium and high salinity conditions. Similarly, salinity stress decreased the relative abundance of *Acidobacteriota* in the no fertilizer treatment, from 5.04 ± 0.24 in control salinity to 3.81 ± 0.44 in medium salinity.

Vermicompost significantly increased the abundance of *Bacteroidota* under both medium and high salinity conditions (*p* < 0.001), while decreasing *Chloroflexota* compared to treatments without fertilizer (*p* < 0.05).

The use of NPK fertilizer had varied effects on bacterial community composition. It significantly reduced the relative abundance of several phyla, including *Planctomycetota* (*p* < 0.001) and *Actinomycetota* under both salinity conditions (*p* < 0.05), and *Acidobacteriota* particularly under control salinity (*p* < 0.001). Conversely, NPK fertilizer enhanced other bacterial groups, significantly increasing *Pseudomonadota* under control salinity (*p* < 0.001), *Bacteroidota* under high salinity (*p* < 0.001), and *Gemmatimonadota* under both control and medium salinity conditions (*p* < 0.001).

LEFSe analysis identified enriched families under different salinity gradients, with high salinity treatments featuring *Oxalobacteraceae, Pseudomonadaceae, Sphingobacteriaceae, Flavobacteriaceae*, and *Rhodobacteraceae* as the top biomarkers. Fertilizers’ treatments enriched different families, with *Xanthomonadaceae, Streptomycetaceae, Microbacteriaceae, Nitrosomonadaceae*, and *Sphingobacteriaceae* in inorganic fertilizer-treated soils, and *Nitrososphaeraceae, Cytophagaceae, Flavobacteriaceae, Pirellulaceae, Nocardiaceae* in vermicompost-treated soils (Supplementary Figure S3).

**FIGURE 5:**
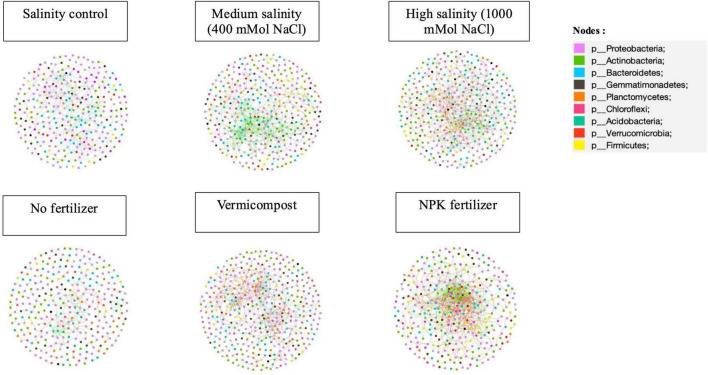
Co-occurring network of bacterial community in response to different salinity and fertilizer treatments for the long-term incubation experiment. Nodes indicate the phyla involved in the networks, and the links indicates the relationship between the different nodes. Green lines represent significant positive relationship, and red represents negative relationships. Each colored dot represents a different phylum.

Salinity increased the network complexity by increasing the number of nodes and edges (Figure 5; Table 2). Furthermore, salinity promoted the network’s positive co-occurrence patterns, especially in medium salinity (75%) (Table 2). Additionally, medium salinity treatments displayed the lowest modularity, the highest average clustering coefficient, and density (Table 2).

**TABLE 2:** Bacterial network properties under different salinity levels and fertilizer treatments for the long-term incubation experiment.

	Treatments	Number of nodes	Number of total links	Number of positive links (%)	Number of negative links	Average clustering coefficient	Density	Modularity
Salinity level	Control salinity	309	438	63.24	36.76%	0.235	0.009	0.78
	Medium salinity^†^	380	688	74.71	25.29	0.242	0.01	0.672
	High salinity^‡^	396	626	51.44	48.56%	0.179	0.008	0.723
Fertilizer type	No fertilizer	309	305	59.34	40.66%	0.173	0.006	0.844
	Vermicompost	418	678	47.49	52.51%	0.236	0.008	0.765
	NPK fertilizer	359	870	51.15	48.58	0.209	0.014	0.528

**^†^**Medium salinity: 0.4 Mol NaCl. **^‡^**High salinity: 1 Mol NaCl.

The use of both fertilizers increased the complexity of the network, manifested by a high number of edges and nodes (Figure 5; Table 2). The use of fertilizers decreased the network modularity, especially in inorganic fertilizer treatments (Table 2).

### 3.2 Impact of salinity and fertilizers application on soil multifunctionality

#### 3.2.1 Short-term incubation experiment

**FIGURE 6:**
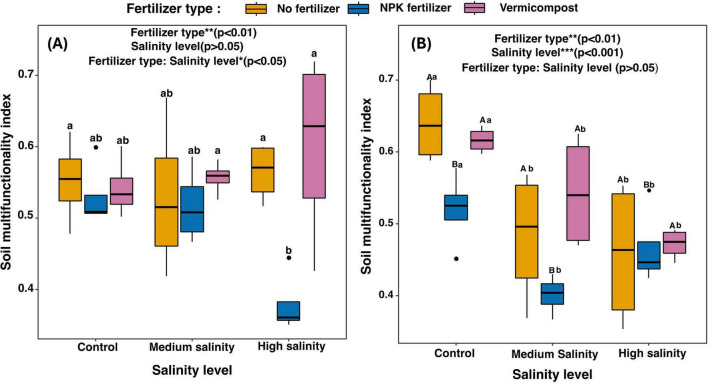
Changes in potential soil multifunctionality of different fertilizer treatments across salinity gradient, **(A)** in the short-term incubation experiment (2 h), **(B)** in the long-term incubation experiment (70 D). The results of the two-way ANOVA are presented at the top of the graph. **(A)** For each combination of fertilizer type and salinity level, means followed by the same lowercase letters are not significantly different at *p* < 0.05. **(B)** For each fertilizer treatment (across salinity levels): means with the same uppercase letters are not significantly different (*p* < 0.05). For each salinity treatment (across fertilizer types): means with the same lowercase letters are not significantly different (*p* < 0.05).

Soil multifunctionality was significantly affected by the fertilizer type (*p* < 0.01) and the interaction of fertilizer type and salinity level (*p* < 0.05) (Figure 6A). Specifically, soil multifunctionality decreased when inorganic fertilizer was applied in high salinity by 34% compared to the no fertilizer treatment.

#### 3.2.2 Long term incubation experiment

Soil multifunctionality showed a significant decrease with increasing salinity by 21.48% (*p* < 0.001). Application of inorganic fertilizer also significantly decreased soil multifunctionality by 12% (*p* < 0.01) (Figure 6B).

**FIGURE 7:**
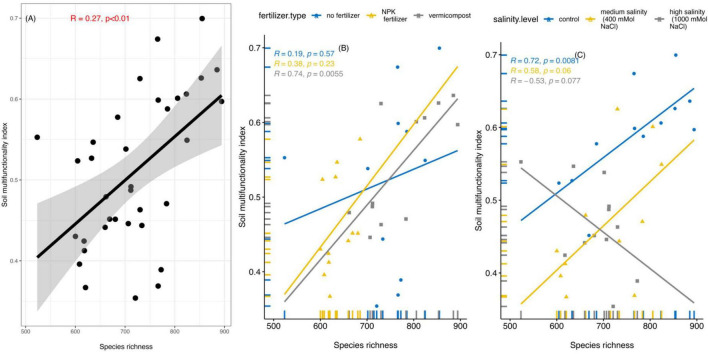
**(A)** Linear regression showing the association between bacterial richness and potential soil multifunctionality for the long-term incubation experiment. Linear regression between bacterial richness and potential soil multifunctionality in different fertilizer treatments **(B)**, and salinity levels **(C)**.

The soil multifunctionality correlated positively with bacterial richness (Figure 7A), and the correlation was strongest in the vermicompost treatments (Figure 7B, *R*^2^ = 0.74, *p* < 0.01). Bacterial richness increased with higher soil multifunctionality across all salinity levels except for the high salinity treatment, where the correlation was negative (Figure 7C, *R*^2^ = –0.53).

## 4 Discussion

Soil salinization is a global challenge that mainly affects arid and semi-arid regions, posing a significant threat to global food security. Our findings emphasize the crucial role of soil management practices in shaping microbial community dynamics and modulating their response to salinity stress. The results of this study highlighted the positive impact of organic fertilizers on improving the soil microbiome’s resilience to salinity stress as it increased microbial biomass, diversity, network complexity and soil multifunctionality. Additionally, this study reveals the negative impact of inorganic fertilizer use as it challenges the ability of the soil microbiome to withstand and recover from salinity stress by reducing microbial diversity and soil multifunctionality.

### 4.1 Salinity produces fast changes in the microbial community structure and function

We hypothesized that exposure to salinity stress would alter the microbial community in the short term. Our findings confirmed this hypothesis: we observed a shift in microbial structure, manifested by a decrease in the bacterial and fungal PLFAs, indicating an immediate stress response affecting microbial biomass and cell membrane integrity (Rath et al., 2017). However, 16S rRNA sequencing did not show significant changes in alpha or beta diversity within this short time frame. This discrepancy can be attributed to the differences in sensitivity and temporal dynamics of the two methods. The PLFA analysis is highly sensitive to rapid physiological changes (Ramsey et al., 2006), reflecting immediate microbial stress responses through alterations in lipid content. In contrast, 16S rRNA sequencing provides a snapshot of community composition and diversity (Orwin et al., 2018), which may require more time to show detectable changes. Thus, highlighting the importance of using complementary approaches to capture both immediate physiological responses (PLFA) and community shifts (16S rRNA) (Lewe et al., 2021). Furthermore, while 16S rRNA gene sequencing detects both active and inactive bacteria, it may not capture rapid functional changes in the soil microbiome. Analyzing the soil metatranscriptome using RNA-based approaches would be necessary to observe and confirm these dynamic shifts.

The introduction of vermicompost altered the microbial community by enhancing bacterial and fungal PLFAs and altering the bacterial community distribution. This indicates immediate microbial activation and decomposition of organic matter, highlighting vermicompost’s positive role in promoting microbial activity and overall soil health. The decrease in the saturated to unsaturated PLFAs ratio following vermicompost application suggests an increase in microbial activity in the short term. This shift indicates that vermicompost supports the growth of microbes with more unsaturated fatty acids, typically associated with enhanced membrane fluidity and higher metabolic activity (Bai et al., 2017). Furthermore, vermicompost provides organic matter, creating a favorable environment for copiotrophic and heterotrophic bacteria (Hollister et al., 2010). For instance, the relative abundance of *Bacteriodota* increased substantially following the application of vermicompost. Additionally, vermicompost use enriched specific bacterial families, such as *Mycobacteriaceae* and *Chitinophagaceae*, known as copiotrophs (Finn et al., 2021; Rath et al., 2019), further emphasizing the pulse effect induced by vermicompost. The presence of these phyla in the soil enhances nutrient cycling, organic matter decomposition (Cusack et al., 2010) and promotes the production of osmolytes that can help maintain cellular functions under saline conditions, improving the soil resilience to salinity stress.

While the application of vermicompost promoted a shift toward a more copiotrophic community within just 2 h, the bacterial community structure remained unchanged with the use of inorganic fertilizer. This discrepancy can be attributed to the soil microbiome’s preference for the organic substrates present in vermicompost (Wang et al., 2022). Furthermore, the use of inorganic fertilizer decreased soil multifunctionality under high salinity stress, suggesting a negative impact on the overall functioning of the soil ecosystem. Such changes could lead to decreased soil fertility (Chen et al., 2022) and increased environmental risks (Delgado-Baquerizo et al., 2016).

### 4.2 Long-term salinity stress challenges soil microbiome resilience: vermicompost enhances recovery while NPK fertilization compromises it

Contrary to our hypothesis, the microbial community remained disturbed by salinity stress after 70 days, suggesting that the microbial community’s resilience to salinity stress was challenged. The exposure to salinity likely caused irreversible shifts in community composition through strong environmental filtering that favored the presence of salt-tolerant taxa while eliminating sensitive ones (Rath et al., 2019). Additionally, the high energetic costs of osmotic adaptation to salinity may have depleted resources needed for microbial population recovery (Griffiths and Philippot, 2013). Furthermore, salinity stress can shift the soil microbial communities into a new alternative stable state (Vrieze et al., 2017).

Salinity stress significantly altered the beta diversity of the bacterial community, as evidenced by distinct clustering patterns in PCoA. It also reduced bacterial richness, causing a decline in soil multifunctionality, as confirmed by Wagg et al. (2019) and Cheng and Wan (2023). Additionally, salinity altered the relative abundance of bacterial phyla. For instance, the relative abundance of *Acidobacteriota* and *Planctomycetota* decreased with increasing salinity. This decline can be attributed to their sensitivity to osmotic stress and lack of salt tolerance mechanisms, making them less competitive under salinity conditions (Yang et al., 2020). In contrast, *Bacteroidota*, a phylum that includes salt-tolerant and halophilic bacteria, increased in abundance (Canfora et al., 2014; Dong et al., 2022; Hou et al., 2021; Rath et al., 2019; Zhang et al., 2019).

In agreement with our hypothesis, the fertilizers had varying effects on the soil microbial community in the long-term. Applying inorganic fertilizer increased the bacterial PLFA groups in medium salinity but decreased them in high salinity. This initial increase can be attributed to the immediate availability of nutrients provided by the fertilizer, which can stimulate microbial growth and activity. However, at high salinity levels, the benefits of inorganic fertilizer appear to diminish. This could be due to the increased osmotic stress imposed by high salinity, which can damage microbial cells and reduce their activity (She et al., 2021).

In contrast, applying vermicompost increased the bacterial PLFA groups even in high salinity conditions. Vermicompost provides not only essential nutrients but also organic matter, which can improve soil health, thereby mitigating the adverse effects of salinity and allowing for better resilience to salinity (Gu et al., 2019; Hubanks et al., 2018).

On the other hand, the 16S rRNA sequencing revealed that the use of NPK fertilizer reduced alpha diversity and bacterial richness as observed by Gao et al. (2019), Gu et al. (2019), and Wang et al. (2023) causing a decline in soil multifunctionality. This reduction in diversity and richness with NPK fertilizer could be due to the selective pressure exerted by the readily available inorganic nutrients, which may favor the growth of certain bacterial taxa over others, leading to a less diverse microbial community (Dai et al., 2018). In contrast, vermicompost, with its diverse array of nutrients and organic matter, supports a more balanced microbial environment, allowing for the enhancement of bacterial diversity (Maji et al., 2017). A study by Mao et al. (2022), demonstrated that applying organic amendments enhances bacterial diversity, leading to a shift in the soil microbial community toward a more salt-tolerant bacterial population (Sall et al., 2015). Indeed, the use of vermicompost-enriched *Mycobacteriaceae*, commonly found in saline environments (Rath et al., 2019), as well as the *Gordoniaceae* family, known for its salt-tolerant species (Kayasth et al., 2014). Additionally, *Mycobacteriaceae*, which belongs to the *Actinomycetota* phylum, are recognized for their adaptation to stress (Kim and Lee, 2014). Moreover, the *Mycobaceriaceae* family possesses genes involved in glutamate synthesis and produces osmolytes to regulate the osmotic pressure (Finn et al., 2021).

While vermicompost did not directly increase soil multifunctionality in this study, numerous other studies have demonstrated the positive impact of organic fertilizers on enhancing soil multifunctionality. Song et al. (2024) confirmed that using organic amendment improved soil ecosystem multifunctionality in saline soils. A study by Jia et al. (2023) revealed that the decrease in soil salinization, along with the increase in the soil organic matter amount, is linked to the enhancement of soil multifunctionality by promoting the diversity and abundance of bacterial community. Additionally, Tang et al. (2023) found that organic fertilizer substitutions improved soil multifunctionality by enhancing bacterial network complexity and improving bacterial diversity.

Co-occurrence analysis showed that increasing salinity increased the complexity of the network, with the medium salinity treatment displaying the most complex system in terms of the high number of nodes and edges. This aligns with findings by Dong et al. (2022), who reported that severe salinity increased network complexity, possibly as an adaptive response to sudden salinity changes, maintaining ecosystem functions. The high percentage of positive links suggests increased cooperation among bacteria to mitigate salt stress, emphasizing their coexistence and interdependence. However, a high number of positive links may compromise the stability of the bacterial network. This is attributed to the formation of positive feedback loops, where a reduction in the abundance of taxa within the loop adversely impacts other taxa within the same loop (Hernandez et al., 2021). Additionally, the medium salinity treatment had a low modularity, indicating a decrease in the stability of the bacteria network following salinity stress (Hernandez et al., 2021).

Using both fertilizers led to a more complex system, as the number of nodes and edges was high when compared to the control. However, vermicompost treatments displayed a more stable network as the modularity was higher than inorganic fertilizer treatments. Mao et al. (2022) also noted that various organic fertilizers enhanced microbial network complexity and stability. Although a more complex network is typically linked to greater stability (Yuan et al., 2021), our findings indicate the contrary for NPK fertilizer treatment. This treatment exhibits a more complex network compared to vermicompost treatments, yet it is less stable. This may be attributed to reduced functional redundancy from decreased bacterial diversity after applying NPK fertilizer (Li et al., 2022; Zhou et al., 2022), resulting in a less stable system. In contrast, organic amendments have the potential to increase microbial diversity and functional redundancy (Farrell et al., 2009), resulting in a more stable system and more resilient to abiotic stressors.

Compared to vermicompost application, the co-occurrence network analysis results align with PLFA and PcoA results, emphasizing the disruptive impact of inorganic fertilizer on the soil microbial community. Thus, it will be more challenging for the microbiome community to recover from the salinity stress when inorganic fertilizer is applied.

## 5 Conclusion

This study shows the change in soil microbial function following short-term exposure to salinity stress. However, the microbiome structure demonstrates resistance to the immediate effects of salinity. Over a 70-day incubation period, the soil microbiome did not recover from the impact of salinity as expected. On the contrary, salinity stress induces a shift in the microbial community’s composition, reducing bacterial diversity and soil multifunctionality and enhancing the complexity of the bacterial network. The addition of vermicompost enhances bacterial richness, favors the growth of salt-tolerant bacteria, and inhibits the effect of salinity on soil multifunctionality. In contrast, inorganic fertilizer use reduces bacterial richness, diversity, and soil multifunctionality. The findings of this study highlight the importance of using vermicompost to alleviate the adverse effects of salinity on the soil microbiome. It also sheds light on the negative impacts of inorganic fertilizers, which can worsen the effects of salinity stress on the soil microbiome and hinder its recovery.

## Data Availability

The data presented in the study are deposited in the NCBA Sequence Read Archive repository, accession number PRJNA1270036.

## References

[B1] Abdul Rahman N. S. N. Abdul Hamid N. W. Nadarajah K. (2021). Effects of abiotic stress on soil microbiome. *Int. J. Mol. Sci.* 22:9036. 10.3390/ijms22169036 34445742 PMC8396473

[B2] Ait-El-Mokhtar M. Baslam M. Ben-Laouane R. Anli M. Boutasknit A. Mitsui T. (2020). Alleviation of detrimental effects of salt stress on date palm (*Phoenix dactylifera* L.) by the application of arbuscular mycorrhizal fungi and/or compost. *Front. Sustain. Food Syst.* 4:131. 10.3389/fsufs.2020.00131

[B3] Bai Z. Ma Q. Wu X. Zhang Y. Yu W. (2017). Temperature sensitivity of a PLFA-distinguishable microbial community differs between varying and constant temperature regimes. *Geoderma* 308, 54–59. 10.1016/j.geoderma.2017.08.026

[B4] Bell C. W. Fricks B. E. Rocca J. D. Steinweg J. M. McMahon S. K. Wallenstein M. D. (2013). High-throughput fluorometric measurement of potential soil extracellular enzyme activities. *JVE* 81:e50961. 10.3791/50961 24299913 PMC3991303

[B5] Benazzouk S. Dobrev P. I. Djazouli Z.-E. Motyka V. Lutts S. (2020). Positive impact of vermicompost leachate on salt stress resistance in tomato (*Solanum lycopersicum* L.) at the seedling stage: A phytohormonal approach. *Plant Soil* 446 145–162. 10.1007/s11104-019-04361-x

[B6] Bolyen E. Rideout J. R. Dillon M. R. Bokulich N. A. Abnet C. C. Al-Ghalith G. A. (2019). Reproducible, interactive, scalable and extensible microbiome data science using QIIME 2. *Nat. Biotechnol.* 37 852–857. 10.1038/s41587-019-0209-9 31341288 PMC7015180

[B7] Bursy J. Kuhlmann A. U. Pittelkow M. Hartmann H. Jebbar M. Pierik A. J. (2008). Synthesis and uptake of the compatible solutes ectoine and 5-hydroxyectoine by *Streptomyces coelicolor* A3(2) in response to salt and heat stresses. *Appl. Environ. Microbiol.* 74 7286–7296. 10.1128/AEM.00768-08 18849444 PMC2592907

[B8] Buyer J. S. Vinyard B. Maul J. Selmer K. Lupitskyy R. Rice C. (2019). Combined extraction method for metabolomic and PLFA analysis of soil. *Appl. Soil Ecol*. 135, 129–136. 10.1016/j.apsoil.2018.11.012

[B9] Byrnes J. E. K. Gamfeldt L. Isbell F. Lefcheck J. S. Griffin J. N. Hector A. (2014). Investigating the relationship between biodiversity and ecosystem multifunctionality: Challenges and solutions. *Methods Ecol. Evol.* 5 111–124. 10.1111/2041-210X.12143

[B10] Canfora L. Bacci G. Pinzari F. Lo Papa G. Dazzi C. Benedetti A. (2014). Salinity and bacterial diversity: To what extent does the concentration of salt affect the bacterial community in a saline soil? *PLoS One* 9:e106662. 10.1371/journal.pone.0106662 25188357 PMC4154724

[B11] Castellano-Hinojosa A. Strauss S. L. González-López J. Bedmar E. J. (2021). Changes in the diversity and predicted functional composition of the bulk and rhizosphere soil bacterial microbiomes of tomato and common bean after inorganic N-fertilization. *Rhizosphere* 18:100362. 10.1016/j.rhisph.2021.100362

[B12] Chen G.-X. Wu C.-F. Ge T. Chen J.-P. Deng Y.-W. (2022). Response of soil multifunctionality to reduced microbial diversity. *Huan Jing Ke Xue* 43 5274–5285. 10.13227/j.hjkx.202201095 36437099

[B13] Cheng Y. Wan W. (2023). Elevated salinity decreases soil ecosystem multifunctionality by shifting the bacterial community from K- to r-selected living strategy. *Land Degrad. Dev.* 34 1110–1119. 10.1002/ldr.4519

[B14] Cusack D. F. Torn M. S. McDOWELL W. H. Silver W. L. (2010). The response of heterotrophic activity and carbon cycling to nitrogen additions and warming in two tropical soils. *Glob. Change Biol.* 16 2555–2572. 10.1111/j.1365-2486.2009.02131.x

[B15] Dai Z. Su W. Chen H. Barberán A. Zhao H. Yu M. (2018). Long-term nitrogen fertilization decreases bacterial diversity and favors the growth of Actinobacteria and Proteobacteria in agro-ecosystems across the globe. *Glob. Change Biol*. 24, 3452–3461. 10.1111/gcb.14163 29645398

[B16] Delgado-Baquerizo M. Maestre F. T. Reich P. B. Jeffries T. C. Gaitan J. J. Encinar D. (2016). Microbial diversity drives multifunctionality in terrestrial ecosystems. *Nat. Commun.* 7:10541. 10.1038/ncomms10541 26817514 PMC4738359

[B17] Dhariwal A. Chong J. Habib S. King I. L. Agellon L. B. Xia J. (2017). MicrobiomeAnalyst: A web-based tool for comprehensive statistical, visual and meta-analysis of microbiome data. *Nucleic Acids Res.* 45 W180–W188. 10.1093/nar/gkx295 28449106 PMC5570177

[B18] Doane T. A. Horwath W. R. (2003). Spectrophotometric determination of nitrate with a single reagent. *Anal. Lett.* 36 2713–2722. 10.1081/AL-120024647

[B19] Dong Y. Zhang J. Chen R. Zhong L. Lin X. Feng Y. (2022). Microbial community composition and activity in saline soils of coastal agro–ecosystems. *Microorganisms* 10:835. 10.3390/microorganisms10040835 35456884 PMC9027772

[B20] Farrell M. Griffith G. W. Hobbs P. J. Perkins W. T. Jones D. L. (2009). Microbial diversity and activity are increased by compost amendment of metal-contaminated soil. *FEMS Microbiol. Ecol.* 71 94–105. 10.1111/j.1574-6941.2009.00793.x 19845764

[B21] Feng K. Peng X. Zhang Z. Gu S. He Q. Shen W. (2022). iNAP: An integrated network analysis pipeline for microbiome studies. *iMeta* 1:e13. 10.1002/imt2.13 38868563 PMC10989900

[B22] Fierer N. Wood S. A. Bueno de Mesquita C. P. (2021). How microbes can, and cannot, be used to assess soil health. *Soil Biol. Biochem.* 153:108111. 10.1016/j.soilbio.2020.108111

[B23] Finn D. R. Bergk-Pinto B. Hazard C. Nicol G. W. Tebbe C. C. Vogel T. M. (2021). Functional trait relationships demonstrate life strategies in terrestrial prokaryotes. *FEMS Microbiol. Ecol.* 97:fiab068. 10.1093/femsec/fiab068 33960387

[B24] Frostegård A. Bååth E. (1996). The use of phospholipid fatty acid analysis to estimate bacterial and fungal biomass in soil. *Biol. Fertil. Soils* 22 59–65. 10.1007/BF00384433

[B25] Gao Y. Sun S. Xing F. Mu X. Bai Y. (2019). Nitrogen addition interacted with salinity-alkalinity to modify plant diversity, microbial PLFAs and soil coupled elements: A 5-year experiment. *Appl. Soil Ecol.* 137 78–86. 10.1016/j.apsoil.2019.01.011

[B26] Garcia-Franco N. Wiesmeier M. Colocho Hurtarte L. C. Fella F. Martínez-Mena M. Almagro M. (2021). Pruning residues incorporation and reduced tillage improve soil organic matter stabilization and structure of salt-affected soils in a semi-arid Citrus tree orchard. *Soil Tillage Res.* 213:105129. 10.1016/j.still.2021.105129

[B27] Gravuer K. Guanasekara A. (2016). *Compost application rates for California croplands and rangelands for a CDFA healthy soils incentives program.* California, CA: California Department of Food and Agriculture.

[B28] Griffiths B. S. Philippot L. (2013). Insights into the resistance and resilience of the soil microbial community. *FEMS Microbiol. Rev.* 37 112–129. 10.1111/j.1574-6976.2012.00343.x 22568555

[B29] Gu S. Hu Q. Cheng Y. Bai L. Liu Z. Xiao W. (2019). Application of organic fertilizer improves microbial community diversity and alters microbial network structure in tea (*Camellia sinensis*) plantation soils. *Soil Tillage Res.* 195:104356. 10.1016/j.still.2019.104356

[B30] Haj-Amor Z. Araya T. Kim D.-G. Bouri S. Lee J. Ghiloufi W. (2022). Soil salinity and its associated effects on soil microorganisms, greenhouse gas emissions, crop yield, biodiversity, and desertification: A review. *Sci. Total Environ.* 843:156946. 10.1016/j.scitotenv.2022.156946 35768029

[B31] Haney R. L. Haney E. B. (2010). Simple and rapid laboratory method for rewetting dry soil for incubations. *Commun. Soil Sci. Plant Anal.* 41 1493–1501. 10.1080/00103624.2010.482171

[B32] Hernandez D. J. David A. S. Menges E. S. Searcy C. A. Afkhami M. E. (2021). Environmental stress destabilizes microbial networks. *ISME* 15 1722–1734. 10.1038/s41396-020-00882-x 33452480 PMC8163744

[B33] Hollister E. B. Engledow A. S. Hammett A. J. M. Provin T. L. Wilkinson H. H. Gentry T. J. (2010). Shifts in microbial community structure along an ecological gradient of hypersaline soils and sediments. *ISME* 4 829–838. 10.1038/ismej.2010.3 20130657

[B34] Horwath W. R. Paul E. A. (1994). “Microbial biomass,” in *Methods of soil analysis*, eds Weaver R. W. Angle J. S. Bottomley P. J. Bezdicek D. F. Smith M. S. Tabatabai M. A. (Hoboken, NJ: John Wiley & Sons, Ltd).

[B35] Hou Y. Zeng W. Hou M. Wang Z. Luo Y. Lei G. (2021). Responses of the soil microbial community to salinity stress in maize fields. *Biology* 10:1114. 10.3390/biology10111114 34827107 PMC8614889

[B36] Hubanks H. L. Deenik J. L. Crow S. E. (2018). “Getting the dirt on soil health and management,” in *Reference module in earth systems and environmental sciences*, ed. S. A. Elias (Amsterdam: Elsevier), 10.1016/B978-0-12-409548-9.10903-0

[B37] Ji L. Yang Y. Yang L. (2021). Seasonal variations in soil fungal communities and co-occurrence networks along an altitudinal gradient in the cold temperate zone of China: A case study on Oakley Mountain. *CATENA* 204:105448. 10.1016/j.catena.2021.105448

[B38] Jia J. Zhang J. Li Y. Koziol L. Podzikowski L. Delgado-Baquerizo M. (2023). Relationships between soil biodiversity and multifunctionality in croplands depend on salinity and organic matter. *Geoderma* 429:116273. 10.1016/j.geoderma.2022.116273

[B39] Jiang S.-Q. Yu Y.-N. Gao R.-W. Wang H. Zhang J. Li R. (2019). High-throughput absolute quantification sequencing reveals the effect of different fertilizer applications on bacterial community in a tomato cultivated coastal saline soil. *Sci. Total Environ.* 687 601–609. 10.1016/j.scitotenv.2019.06.105 31220714

[B40] Kaehler B. D. Bokulich N. A. McDonald D. Knight R. Caporaso J. G. Huttley G. A. (2019). Species abundance information improves sequence taxonomy classification accuracy. *Nat. Commun.* 10:4643. 10.1038/s41467-019-12669-6 31604942 PMC6789115

[B41] Kayasth M. Kumar V. Gera R. (2014). Gordonia sp.: A salt tolerant bacterial inoculant for growth promotion of pearl millet under saline soil conditions. *3 Biotech* 4 553–557. 10.1007/s13205-013-0178-5 28324383 PMC4162897

[B42] Kim K. K. Lee J.-S. (2014). “The family nakamurellaceae,” in *The prokaryotes: Actinobacteria*, eds Rosenberg E. DeLong E. F. Lory S. Stackebrandt E. Thompson E. (Berlin: Springer).

[B43] Lewe N. Hermans S. Lear G. Kelly L. T. Thomson-Laing G. Weisbrod B. (2021). Phospholipid fatty acid (PLFA) analysis as a tool to estimate absolute abundances from compositional 16S rRNA bacterial metabarcoding data. *J. Microbiol. Methods* 188:106271. 10.1016/j.mimet.2021.106271 34146605

[B44] Li X. Li B. Chen L. Liang J. Huang R. Tang X. (2022). Partial substitution of chemical fertilizer with organic fertilizer over seven years increases yields and restores soil bacterial community diversity in wheat–rice rotation. *Eur. J. Agronomy* 133:126445. 10.1016/j.eja.2021.126445

[B45] Luo J. Liao G. Banerjee S. Gu S. Liang J. Guo X. (2023). Long-term organic fertilization promotes the resilience of soil multifunctionality driven by bacterial communities. *Soil Biol. Biochem.* 177:108922. 10.1016/j.soilbio.2022.108922

[B46] Maji D. Misra P. Singh S. Kalra A. (2017). Humic acid rich vermicompost promotes plant growth by improving microbial community structure of soil as well as root nodulation and mycorrhizal colonization in the roots of *Pisum sativum*. *Appl. Soil Ecol.* 110 97–108. 10.1016/j.apsoil.2016.10.008

[B47] Mao X. Yang Y. Guan P. Geng L. Ma L. Di H. (2022). Remediation of organic amendments on soil salinization: Focusing on the relationship between soil salts and microbial communities. *Ecotoxicol. Environ. Saf.* 239:113616. 10.1016/j.ecoenv.2022.113616 35588623

[B48] Miranda K. M. Espey M. G. Wink D. A. (2001). A rapid, simple spectrophotometric method for simultaneous detection of nitrate and nitrite. *Nitric Oxide* 5 62–71. 10.1006/niox.2000.0319 11178938

[B49] Ngosong C. Jarosch M. Raupp J. Neumann E. Ruess L. (2010). The impact of farming practice on soil microorganisms and arbuscular mycorrhizal fungi: Crop type versus long-term mineral and organic fertilization. *Appl. Soil Ecol.* 46 134–142. 10.1016/j.apsoil.2010.07.004

[B50] Orwin K. H. Dickie I. A. Holdaway R. Wood J. R. (2018). A comparison of the ability of PLFA and 16S rRNA gene metabarcoding to resolve soil community change and predict ecosystem functions. *Soil Biol. Biochem*. 117, 27–35. 10.1016/j.soilbio.2017.10.036

[B51] Prasertsuk S. Wijitkosum S. (2021). Innovative use of rice husk biochar for rice cultivation in salt-affected soils with alternated wetting and drying irrigation. *Eng. J.* 25 19–32. 10.4186/ej.2021.25.9.19 12383539

[B52] R Core Team (2022). *R: A language and environment for statistical computing.* Vienna: R Foundation for Statistical Computing.

[B53] Ramsey P. W. Rillig M. C. Feris K. P. Holben W. E. Gannon J. E. (2006). Choice of methods for soil microbial community analysis: PLFA maximizes power compared to CLPP and PCR-based approaches. *Pedobiologia* 50, 275–280. 10.1016/j.pedobi.2006.03.003

[B54] Rath K. M. Fierer N. Murphy D. V. Rousk J. (2019). Linking bacterial community composition to soil salinity along environmental gradients. *ISME* 13 836–846. 10.1038/s41396-018-0313-8 30446737 PMC6461869

[B55] Rath K. M. Maheshwari A. Rousk J. (2017). The impact of salinity on the microbial response to drying and rewetting in soil. *Soil Biol. Biochem.* 108 17–26. 10.1016/j.soilbio.2017.01.018

[B56] Rath K. M. Maheshwari A. Bengtson P. Rousk J. (2016). Comparative toxicities of salts on microbial processes in soil. *Appl. Environ. Microbiol.* 82 2012–2020. 10.1128/AEM.04052-15 26801570 PMC4807522

[B57] Sall S. N. Ndour N. Y. B. Diédhiou-Sall S. Dick R. Chotte J.-L. (2015). Microbial response to salinity stress in a tropical sandy soil amended with native shrub residues or inorganic fertilizer. *J. Environ. Manag.* 161 30–37. 10.1016/j.jenvman.2015.06.017 26143083

[B58] Segata N. Izard J. Waldron L. Gevers D. Miropolsky L. Garrett W. S. (2011). Metagenomic biomarker discovery and explanation. *Genome Biol.* 12:R60. 10.1186/gb-2011-12-6-r60 21702898 PMC3218848

[B59] She R. Yu Y. Ge C. Yao H. (2021). Soil texture alters the impact of salinity on carbon mineralization. *Agron* 11:128. 10.3390/agronomy11010128

[B60] Song J. Zhang H. Chang F. Yu R. Wang J. Chen A. (2024). Subsurface organic amendment of a saline soil increases ecosystem multifunctionality and sunflower yield. *Sci. Total Enviro.* 170276. 10.1016/j.scitotenv.2024.170276 38262534

[B61] Szoboszlay M. Näther A. Liu B. Carrillo A. Castellanos T. Smalla K. (2019). Contrasting microbial community responses to salinization and straw amendment in a semiarid bare soil and its wheat rhizosphere. *Sci. Rep.* 9:9795. 10.1038/s41598-019-46070-6 31278291 PMC6611862

[B62] Tang Q. Xia Y. Ti C. Shan J. Zhou W. Li C. (2023). Partial organic fertilizer substitution promotes soil multifunctionality by increasing microbial community diversity and complexity. *Pedosphere* 33 407–420. 10.1016/j.pedsph.2022.06.044

[B63] University of California Davis. *SoilWeb: An online soil survey browser. California Soil Resource Lab.* Available online at: https://casoilresource.lawr.ucdavis.edu/gmap/ (accessed 25 September, 2023).

[B64] Vrieze J. D. Christiaens M. E. R. Walraedt D. Devooght A. Ijaz U. Z. Boon N. (2017). Microbial community redundancy in anaerobic digestion drives process recovery after salinity exposure. *Water Res.* 111 109–117. 10.1016/j.watres.2016.12.042 28063283

[B65] Wagg C. Schlaeppi K. Banerjee S. Kuramae E. E. van der Heijden M. G. A. (2019). Fungal-bacterial diversity and microbiome complexity predict ecosystem functioning. *Nat. Commun.* 10:4841. 10.1038/s41467-019-12798-y 31649246 PMC6813331

[B66] Wang C. Li X. Hu Y. Zheng R. Hou Y. (2023). Nitrogen addition weakens the biodiversity multifunctionality relationships across soil profiles in a grassland assemblage. *Agric. Ecosyst. Environ.* 342:108241. 10.1016/j.agee.2022.108241

[B67] Wang Y. Wilhelm R. C. Swenson T. L. Silver A. Andeer P. F. Golini A. (2022). Substrate utilization and competitive interactions among soil bacteria vary with life-history strategies. *Front. Microbiol.* 13:914472. 10.3389/fmicb.2022.914472 35756023 PMC9225577

[B68] Wichern F. Islam M. R. Hemkemeyer M. Watson C. Joergensen R. G. (2020). Organic amendments alleviate salinity effects on soil microorganisms and mineralisation processes in aerobic and anaerobic paddy rice soils. *Front. Sustain. Food Syst*. 4:30. 10.3389/fsufs.2020.00030

[B69] Yan N. Marschner P. Cao W. Zuo C. Qin W. (2015). Influence of salinity and water content on soil microorganisms. *Int. Soil Water Conserv. Res.* 3 316–323. 10.1016/j.iswcr.2015.11.003

[B70] Yang C. Wang X. Miao F. Li Z. Tang W. Sun J. (2020). Assessing the effect of soil salinization on soil microbial respiration and diversities under incubation conditions. *Applied Soil Ecology* 155:103671. 10.1016/j.apsoil.2020.103671

[B71] Yuan M. M. Guo X. Wu L. Zhang Y. Xiao N. Ning D. (2021). Climate warming enhances microbial network complexity and stability. *Nat. Climate Change* 11 343–348. 10.1038/s41558-021-00989-9

[B72] Zelles L. (1999). Fatty acid patterns of phospholipids and lipopolysaccharides in the characterisation of microbial communities in soil: A review. *Biol. Fertil. Soil.* 29 111–129. 10.1007/s003740050533

[B73] Zhang W. Wang C. Xue R. Wang L. (2019). Effects of salinity on the soil microbial community and soil fertility. *J. Integr. Agric.* 18 1360–1368. 10.1016/S2095-3119(18)62077-5

[B74] Zhou X. Sun H. Heinonsalo J. Pumpanen J. Berninger F. (2022). Microbial biodiversity contributes to soil carbon release: A case study on fire disturbed boreal forests. *FEMS Microbiol. Ecol.* 98:fiac074. 10.1093/femsec/fiac074 35749564 PMC9303362

