# Supplementary material for: Organic and inorganic fertilizers modulate the response of the soil microbiome to salinity stress
Source: Front Microbiol. 2025 Jun 19;16:1551586. doi: 10.3389/fmicb.2025.1551586 (PMC12222182; doi:10.3389/fmicb.2025.1551586)
Supplement: Supplementary file 1 [file Table_1.docx]

**Supplemetary Table 1**: the changes in PLFA ratios; Gram+/Gram-, Fungi: Bacteria, Sat: Unsaturated, for the short-term and long term incubation experiment, Values are averages of 4 replicates ± *sd*. Different letter indicates a significant difference (*P* < 0.05) between sites by Tukey test.

| Treatment | | 2h | **Gram +/Gram-** | **Fungi/Bacteria** | **Sat/Unsaturated** | 70 days | **Gram +/gram** | **Fungi/Bacteria** | **Saturated/unsaturated** |
| --- | --- | --- | --- | --- | --- | --- | --- | --- | --- |
| **Control**  **Salinity** | **No fertilizer** |  | 1.19±0.019 c | 0.16±0.005 a | 2.74±0.20 a |  | 3.24±1.31 ab | 0.09±0.066 a | 7.55±3.94 ab |
|  | **Vermicompost** |  | 1.34±0.23 bc | 0.17±0.03 a | 2.90±0.82 a |  | 4.11±0.37 a | 0.09±0.005 a | 7.80±1.14 a |
|  | **NPK fertilizer** |  | 1.88±0.40 abc | 0.14±0.03 a | 5.23±2.47 a |  | 2.60±1.56 ab | 0.07±0.037 a | 7.19±4.65 abc |
| **Medium salinity^†^** | **No fertilizer** |  | 2.11±0.44 ab | 0.14±0.02 a | 5.17±1.45 a |  | 1.46±0.07 b | 0.16±0.02 a | 3.03± 0.37 c |
|  | **Vermicompost** |  | 1.29±0.11 bc | 0.17±0.03 a | 2.70±0.59 a |  | 1.89±0.67 b | 0.12±0.05 a | 3.56± 1.25 bc |
|  | **NPK fertilizer** |  | 2.05±0.58 ab | 0.18±0.04 a | 5.31±2.76 a |  | 1.72±0.63 b | 0.08±0.02 a | 3.79± 1.39 abc |
| **High salinity^‡^** | **No fertilizer** |  | 2.43±0.30 a | 0.10±0.03 a | 4.75±0.78 a |  | 2.02±0.45 ab | 0.13±0.04 a | 4.57± 0.90 abc |
|  | **Vermicompost** |  | 2.20±0.66 ab | 0.14±0.02 a | 3.41±1.11 a |  | 2.40±0.40 ab | 0.10±0.02 a | 4.42± 0.90 abc |
|  | **NPK fertilizer** |  | 1.55±0.54 abc | 0.12±0.065 a | 3.89±0.70 a |  | 2.18±0.76 ab | 0.10±0.04 a | 5.20± 2.02 abc |

**^†^** : Medium salinity : 0.4 mol NaCl

**^‡^**: High salinity : 1 mol NaCl

**Supplemtary Table 2**: Changes in % relative abundance of the most abundant phyla, two hours after the application of the salinity and fertilizer treatments. Values are averages of 4 replicates ± sd. The results of the two-way ANOVA are presented at the bottom of the table, with significance levels denoted as follows: ***p<0.001, **p<0.01, *p<0.05, and ns not significant.

| **Salinity level** | **Fertilizer type** | **Pseudomonadota** | **Planctomycetota** | **Bacteroidota^§§^** | **Actinomycetota** | **Chloroflexota^§§^** | **Gemmatimonadota** | **Bacillota** | **Acidobacteriota** |
| --- | --- | --- | --- | --- | --- | --- | --- | --- | --- |
| **Control**  **salinity** | **No fertilizer** | 28.44±3.64 | 2.45±0.40 | 6.16±0.69 Ba | 27.35±1.71 | 3.74±0.38Aa | 3.11±0.46 | 18.07±1.89 | 3.32±0.46 |
|  | **NPK fertilizer** | 26.30±2.09 | 2.37±0.27 | 6.33±0.75 Ba | 25.14±2.16 | 3.39±0.29ABa | 2.98±0.33 | 22.20±1.44 | 3.55±0.47 |
|  | **Vermicompost** | 30.27±2.36 | 2.23±0.19 | 7.73±0.67 Aa | 24.74±1.70 | 2.83±0.37Ba | 2.65±0.16 | 18.96±0.50 | 3.09±0.33 |
| **Medium salinity^†^** | **No fertilizer** | 28.41±1.64 | 2.55±0.22 | 6.48±1.07 Ba | 26.35±3.37 | 3.92±0.43Aa | 2.96±0.14 | 17.55±2.86 | 4.07±0.98 |
|  | **NPK fertilizer** | 26.85±0.64 | 2.55±0.24 | 6.28±0.71 aB | 25.07±2.94 | 3.70±0.46ABa | 2.99±0.30 | 21.14±3.70 | 3.71±0.63 |
|  | **Vermicompost** | 27.49±1.00 | 2.62±0.28 | 7.48±1.22 Aa | 28.05±1.59 | 3.61±0.62 Ba | 2.56±0.19 | 17.78±2.90 | 3.35±0.42 |
| **High salinity^‡^** | **No fertilizer** | 26.26±0.81 | 2.62±0.45 | 6.24±0.63 Ba | 28.65±1.80 | 4.38±0.32 Aa | 3.07±0.23 | 18.12±2.77 | 2.83±0.49 |
|  | **NPK fertilizer** | 27.61±1.79 | 2.53±0.35 | 6.88±0.98 aB | 27.05±5.26 | 3.85±0.19ABa | 2.86±0.52 | 17.93±3.87 | 3.47±0.42 |
|  | **Vermicompost** | 28.90±2.68 | 2.64±0.31 | 8.05±2.43 Aa | 23.62±3.64 | 3.35±0.76 Ba | 2.94±0.40 | 19.39±5.26 | 3.92±0.75 |
| **P value** | | | | | | | | | |
| **Fertilizer type** | | 0.06 ns | 0.97 ns | 0.008** | 0.19 ns | 0.002** | 0.08 ns | 0.13 ns | 0.41 ns |
| **Salinity level** | | 0.57 ns | 0.08 ns | 0.74 ns | 0.76 ns | 0.06 ns | 0.65 ns | 0.56 ns | 0.16 ns |
| **Fertilizer type: salinity level** | | 0.22 ns | 0.71 ns | 0.94 | 0.15 ns | 0.74 ns | 0.46 ns | 0.44 ns | 0.44 ns |

**^†^**: Medium salinity: 0.4 mol NaCl

**^‡^**: High salinity: 1 mol NaCl

^§§^: For each fertilizer type treatment, means followed by the same uppercase letters are not significantly different at p<0.05 For each salinity level means followed by the same lowercase letters are not significantly different at p<0.05 according to Tukey test results.

**Supplementary Table 3**: Changes in % relative abundance of the most abundant phyla, 70 days after the application of the salinity and fertilizer treatments. Values are averages of 4 replicates±sd. The results of the two-way ANOVA are presented at the bottom of the table, with significance levels denoted as follows: ***p<0.001, **p<0.01, *p<0.05, and ns not significant.

| **Salinity level** | **Fertilizer type** | **Pseudomonadota^§§^** | **Planctomycetota^§§^** | **Bacteroidota^§^** | **Actinomycetota^§^** | **Chloroflexota^§§^** | **Gemmatimonadota^§^** | **Bacillota** | **Acidobacteriota^§^** |
| --- | --- | --- | --- | --- | --- | --- | --- | --- | --- |
| **Control**  **salinity** | **No fertilizer** | 26.10±0.54 Ba | 3.24±0.18 Aa | 4.47±0.32 cd | 25.54±1.13 abc | 4.62±0.36 Aa | 4.21±0.44 cd | 18.97±0.52 | 5.04±0.24 a |
|  | **NPK fertilizer** | 33.10±2.37 Aa | 2.83±0.18 Ba | 3.75±0.46 d | 22.95±2.95 bc | 4.20±0.32 Aa | 7.67±0.60 a | 19.83±1.70 | 2.03±0.22 e |
|  | **Vermicompost** | 27.02±0.50 Ba | 3.27±0.13 Aa | 4.76±0.17 cd | 23.24±0.45 bc | 3.79±0.27 Ba | 4.65±0.54 c | 19.71±0.53 | 5.27±0.51 a |
| **Medium salinity^†^** | **No fertilizer** | 25.77 ± 0.83 Ba | 3.00±0.24Ab | 4.88±0.83 bc | 29.19±3.10 a | 4.44±0.42 Aa | 4.31±0.40 cd | 16.77±1.03 | 3.81±0.44 b |
|  | **NPK fertilizer** | 35.14±2.86 Aa | 2.57±0.23Bb | 4.67±0.47 cd | 21.34±2.28 bc | 4.44±0.75 Aa | 5.87±0.42 b | 18.52±1.85 | 2.35±0.27de |
|  | **Vermicompost** | 26.00±1.58 Ba | 3.04±0.23 Ab | 5.94±0.48 ab | 26.01±0.87 ab | 4.35±0.34 Ba | 3.51±0.39 cd | 20.36±1.38 | 3.36±0.60 bc |
| **High salinity^‡^** | **No fertilizer** | 25.81±1.02 Ba | 2.72±0.32 Ac | 4.82±0.34cd | 28.72±1.86 a | 4.14±0.46 Aa | 3.59±0.42 cd | 18.76±1.93 | 3.42±0.45 bc |
|  | **NPK fertilizer** | 31.78.65±1.98Aa | 2.09±0.23 Bc | 6.83±0.55 a | 20.92±1.32 c | 4.48±0.58 Aa | 3.73±0.58 cd | 20.28±0.82 | 2.54±0.28 cde |
|  | **Vermicompost** | 26.84±0.49 Ba | 2.67±0.19 Ac | 6.78±0.12 a | 23.38±2.10 bc | 3.52±0.25 Ba | 3.31±0.42 d | 21.42±1.61 | 3.21±0.37 bcd |
| **P value** | | | | | | | | | |
| **Fertilizer type** | | 4.56 × 10⁻¹²*** | 7.34×10^-6^*** | 1.30×10^-5^*** | 2.30×10^-7^*** | 0.01* | 1.51×10^-10^*** | 0.13 ns | 1.27×10^-11^*** |
| **Salinity level** | | 0.43 ns | 1.43×10^-6^*** | 2.23×10^-9^*** | 0.14 ns | 0.15 ns | 7.24×10^-10^*** | 0.36 ns | 7.37×10^-7^*** |
| **Fertilizer type: salinity level** | | 0.11 ns | 0.87 ns | 3.89×10^-5^*** | 0.04* | 0.19 ns | 1.93×10^-6^*** | 0.87 ns | 3.56×10^-6^*** |

**^†^**: Medium salinity: 0.4 mol NaCl

**^‡^**: High salinity: 1mol NaCl

^§^: For each combination of fertilizer type and salinity level, means followed by the same lowercase letters are not significantly different at p<0.05 according to Tukey test results.

^§§^: For each fertilizer type treatment, means followed by the same uppercase letters are not significantly different at p<0.05 For each salinity level means followed by the same lowercase letters are not significantly different at p<0.05 according to Tukey test results.


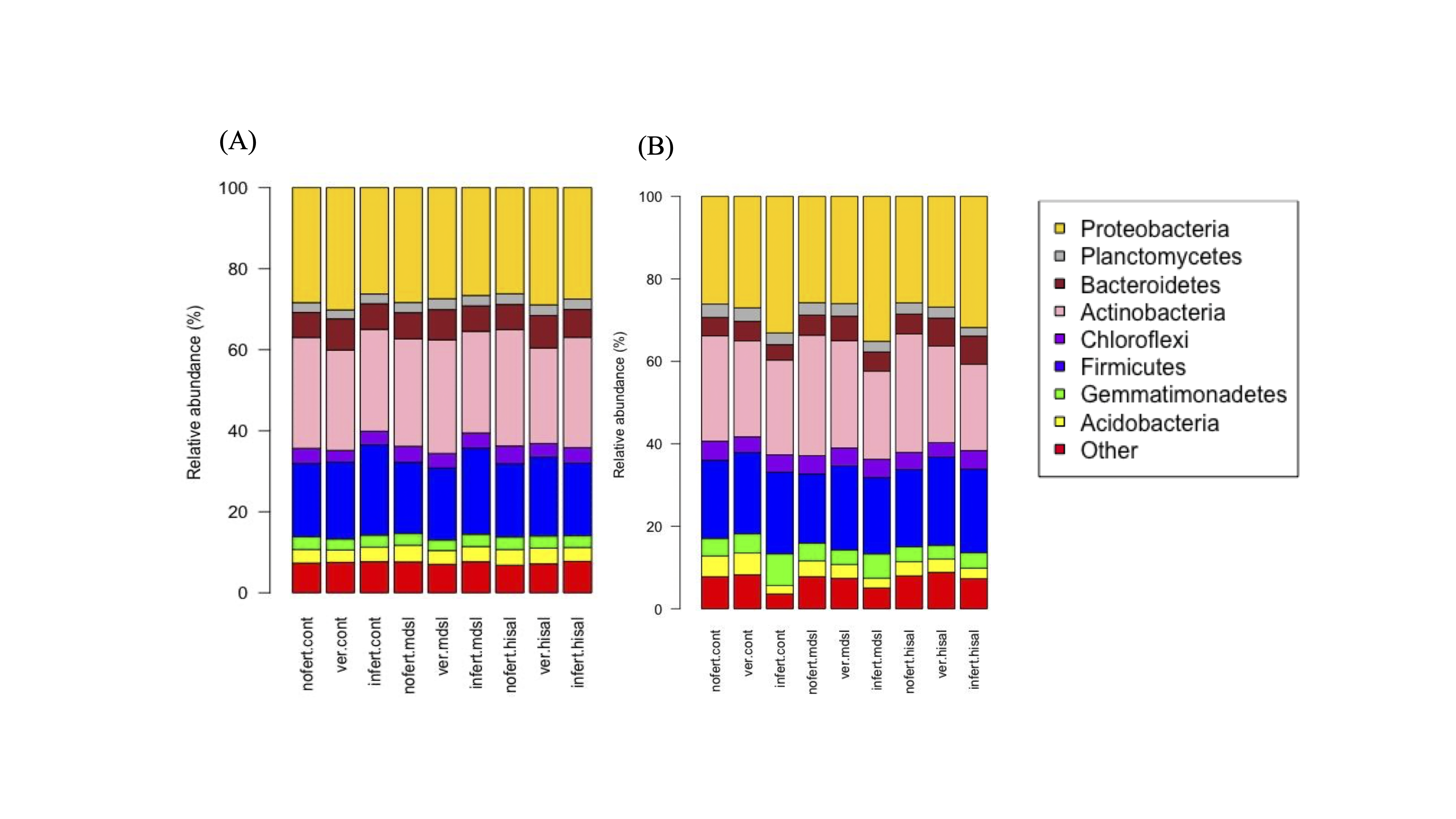


**Supplemntary Figure 1**: Relative abundance of the most abundant prokaryotic phyla across different combinations of salinity level and fertilizer type in the short-term (A), and long-term incubation experiment (B). Treatments: Nofert.cont: No fertilizer control salinity, ver.cont: vermicompost control, infert.cont: inorganic fertilizer control salinity, nofert..mdsl: no fertilizer medium salinity, ver.mdsl: vermicompost medium salinity, infert..mdsl: inorganic fertilizer medium salinity, nofert.hisal: no fertilizer high salinity, ver.hisal: vermicompost high salinity, infert.hisal: inorganic fertilizer high salinity

***
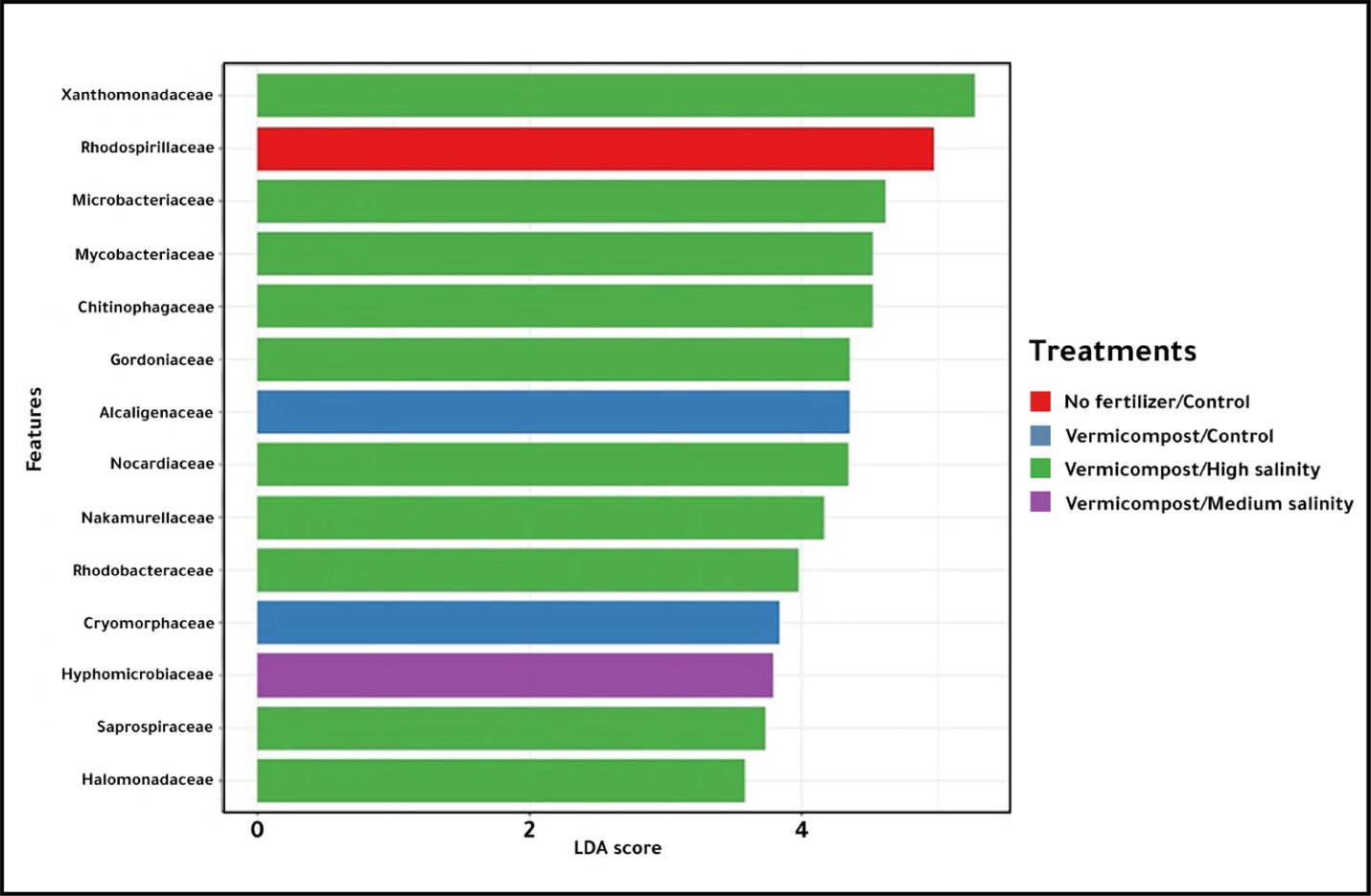
***

**Supplementary Figure 2**: Linear discriminant analysis (LDA) of statistically different families’ abundances between treatments, only families with the highest LDA score and with a significant p value(p<0.05) were presented on the figure.


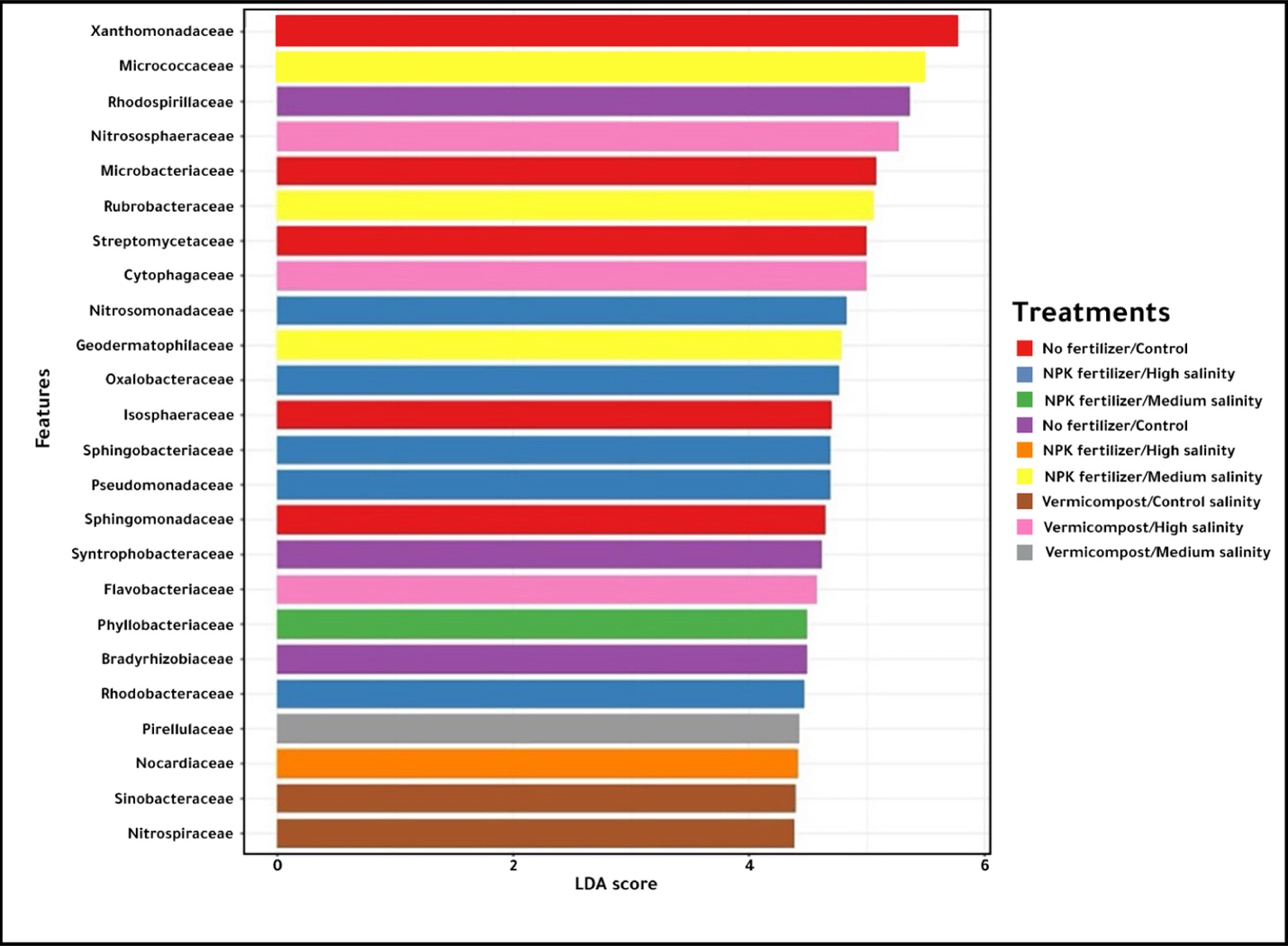


**Supplementray Figure 3:** Linear discriminant analysis (LDA) of statistically different families’ abundances between treatments for the long-term experiment, only families with the highest LDA score and with a significant p value(p<0.05) were presented on the figure.
